# Task force veterinary anatomy: joint efforts of the five German veterinary schools to ensure education during the COVID-19 pandemic

**DOI:** 10.3205/zma001483

**Published:** 2021-06-15

**Authors:** Dora Bernigau, Mahtab Bahramsoltani, Giuliano Mario Corte, Sven Reese, Christiane Pfarrer, Daniela Fietz

**Affiliations:** 1 Leipzig University, Faculty of Veterinary Medicine, Institute of Anatomy, Histology and Embryology, Leipzig, Germany; 2 Freie Universität Berlin, Department of Veterinary Medicine, Institute of Veterinary Anatomy, Berlin, Germany; 3 LMU Munich, Faculty of Veterinary Medicine, Department of Veterinary Sciences, Chair of Anatomy, Histology and Embryology, Munich, Germany; 4 University of Veterinary Medicine Hannover, Foundation, Institute for Anatomy, Hannover, Germany; 5 Justus-Liebig-University Giessen, Institute of Veterinary Anatomy, Histology and Embryology, Giessen, Germany

**Keywords:** anatomy, digital education media, education, veterinary medicine

## Abstract

At the start of the COVID-pandemic in March 2020, the Institutes of Veterinary Anatomy of the five German educational institutions were confronted with the challenge of digitalising all lectures for the second and fourth semesters of veterinary students. After an online kick-off event and a preliminary status quo meeting, available digital teaching material was exchanged for students to stream from learning platforms. Lectures were either synchronized or made available as audio recordings and connotated slides on the learning platforms. Fortunately, digital microscopic slides had already been in use, which made it easy for students to access them. Dissection exercises mostly consisted of self-study, using instructive videos and interactive exercises. In the second half of the semester, four of the educational institutions were able to offer a restricted number of in-person gross anatomy classes under reinforced conditions. Success monitoring took place online through different formats, and partially on a voluntary basis, via the learning platforms. Although the past two semesters had to almost exclusively take place online due to the unprecedented circumstances, and joint efforts of the five veterinary institutions, there is a general consensus that the practical education in anatomy, histology and embryology is essential to veterinary students. In fact, it is the only way they can obtain the necessary skills to successfully complete the rest of their degree.

## Zusammenfassung

Die Veterinäranatomischen Institute der fünf deutschen Bildungsstätten standen zu Beginn der Coronapandemie im März 2020 vor der Herausforderung, die gesamte Lehre für die Studierenden des zweiten und vierten Fachsemesters in digitaler Form durchzuführen. Nach einem Kickoff-Onlinemeeting und einer ersten Bestandsaufnahme wurden vorhandene digitale Lehrmedien zum Streamen auf den Lernplattformen ausgetauscht. Vorlesungen wurden synchron gehalten oder asynchron als vertonte bzw. annotierte Dateien auf den Lernplattformen hochgeladen. Die mikroskopischen Präparate konnten den Studierenden dank schon bestehender Anwendungen zur virtuellen Mikroskopie komplikationslos zur Verfügung gestellt werden. Die Präparierübungen wurden überwiegend zum Selbststudium anhand von Lehrvideos und interaktiven Übungsaufgaben angeboten. In der zweiten Semesterhälfte wurden in der makroskopischen Anatomie an vier Standorten einige wenige Präsenztermine für die Studierenden unter verschärften Hygienebedingungen angeboten. Erfolgskontrollen wurden ausschließlich online über die Lernplattformen in verschiedenen Formaten und zum Teil fakultativ durchgeführt. Auch wenn die vergangenen beiden Semester in Zusammenarbeit aller Standorte den Umständen geschuldet fast ausschließlich digital stattfinden musste, besteht der allgemeine Konsens, dass die praktische Ausbildung in den Fächern Anatomie, Histologie und Embryologie essentiell für die Studierenden der Veterinärmedizin ist. Nur so können sie die notwendigen Kenntnisse und Fertigkeiten für ihr weiteres erfolgreiches Studium erlangen.

## Introduction

In March 2020, a conference of the veterinary anatomists of the educational institutions (Berlin, Giessen, Hannover, Leipzig, Munich) was supposed to take place. The focus was set on the reduction of formalin in veterinary education. Due to the pandemic, the conference was cancelled. Shortly after, the decision was made to hold the conference online with a different main topic. All educational institutions had to cut in-person formats with students from their semester plans and adapt the summer term to teaching without practical classes. This kick-off meeting was followed up by six additional online meetings and an unprecedented in-depth exchange.

## Implementation of anatomy and histology/embryology education in the summer term 2020

### Dissection classes

Conducting dissection classes without being able to teach in-person was the biggest challenge. Just like in human medicine and dentistry, practical education is a fundamental part of anatomical teaching. This is also substantiated by the fact that the licensing ordinance for both human doctors (ÄAppO §22 (2)) and veterinarians (TAppV §24) requires a practical anatomy examination [https://www.gesetze-im-internet.de/_appro_2002/BJNR240500002.html], [https://www.gesetze-im-internet.de/tappv/BJNR182700006.html]. The fourth semester (4. FS) was particularly affected by this problem, since students have to complete their final pre-clinical exams (Physikum) at the end of the term and had not had the opportunity to practice the practical exam contents since February. The status quo exchange showed that all educational institutions already had some digital learning material (video demos, e-lectures, etc.). It was ensured that sharing this content was approved by the responsible parties. This allowed students to stream numerous media from the learning platforms. After the first statutory liberalizations came into effect, and allowed student access to the universities, dissection classes were offered at four of the veterinary educational institutions under strict compliance with the mask-distance-hygiene rules of the Federal German Government and after creating hygiene concepts (defining entrance and exit points, reduction of class size, etc.), for the second (N=1) and the fourth semester (N=3). The conduction of these in-person classes created a significant additional workload for the institutes, while ensuring practical exercise for students with finished specimens. The consistently positive feedback from students confirmed that these classes provided significant benefits for students’ learning success. 

#### Lectures

All lectures at all locations were made accessible digitally, as the university leadership quickly announced that in-person lectures were suspended for the entire academic year due to the ongoing pandemic. Lectures were mainly uploaded to the learning platforms (Blackboard, Moodle and others) as presentations with audio recordings. In addition, at three locations lectures were held synchronously in live sessions via *Zoom, WebEx, MS Team* etc. 

#### Microscopy classes

The entirely digital conduction of this classes was the smallest challenge, as all educational institutions had provided students with virtual microscopy applications prior to the pandemic. This allowed for asynchronous teaching of histological specimens after giving digital instructions. Two faculties offered this class as synchronous online sessions. 

#### Examinations

The regulations for practical classes at all educational institutions include successful participation in different examination formats throughout the semester (mostly oral-practical). Since there were no in-person classes during the summer term 2020, examinations exclusively took place online or were suspended. They were conducted via the learning platforms at four educational institutions. Evaluation modes varied between mandatory participation, cumulative point scores and a binary pass/fail mode. An analysis of the results showed a distribution of scores similar to those of previous terms at all educational institutions. The time to complete the exams was deliberately held short to discourage students from using additional resources.

Students had access to forums regarding all topics on the learning platforms to address any open questions. 

In summer, students in the fourth semester had to complete their final pre-clinical exams (Physikum). According to the TAppV, they have to demonstrate theoretical and practical knowledge of the anatomy on animal bodies. Since these regulations could not be adapted on short notice, the learning objectives of the educational institutions were focussed on practical demonstrations. Since practical exercises were basically non-existent during the fourth semester, these topics were not examined in as much practical detail as they had been in previous years at most locations. Overall, students’ results in the pre-clinical exams are comparable to those of previous years. However, the distribution of grades at four locations indicates that the average grades (between 3 and 4) were less commonly awarded than in previous years, while the grades “good” and “poor” were more commonly achieved. 

#### Creating 3D-Scans for self-study

**Figure 1:**
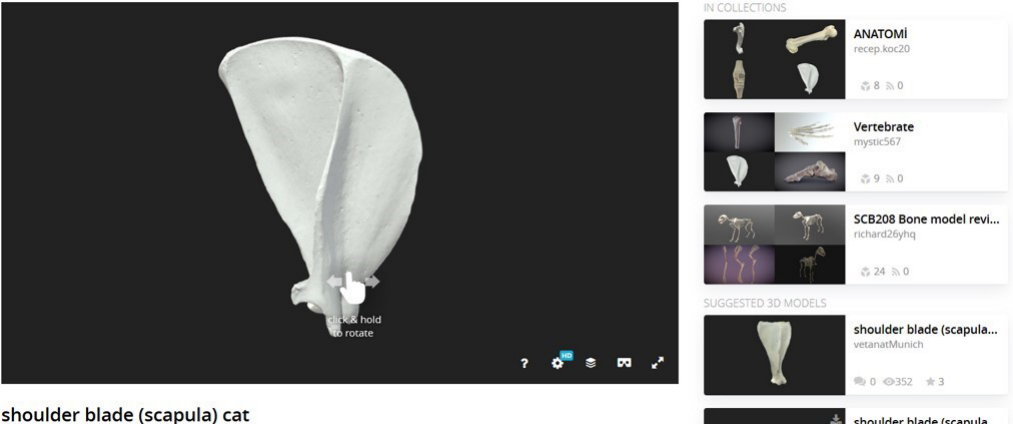
Screenshot from the webpage https://sketchfab.com with a selection of 3D-Scans created by the Chair of Anatomy, Histology and Embryology at the Faculty of Veterinary Medicine of the LMU Munich, Germany.

3D-Scans are a great asset for teaching the three-dimensional understanding of anatomical specimens to students at home. In addition to the 3D-scanners already in use at two educational institutions, one location was able to scan over 700 new specimens within a few months. About 350 of them are now worldwide available at no cost via the URL [https://sketchfab.com/vetanatMunich] (see figure 1 (Fig. 1)) [1].

## Winter term 20201/21 and an outlook to post-COVID-19 times

**Figure 2:**
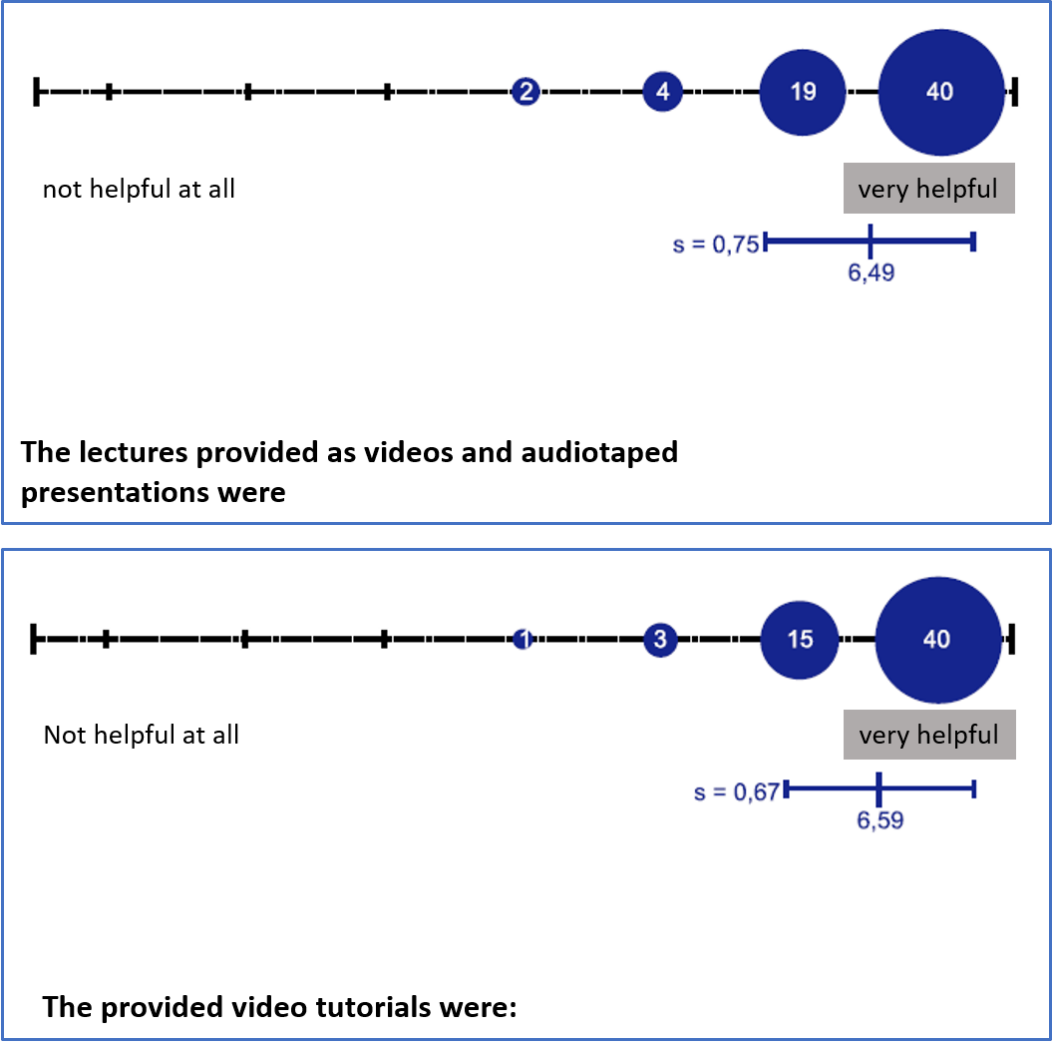
Partial results from student evaluations (N=66, 4^th^ semester) regarding digital teaching material in the summer term 2020, Institute of Veterinary Anatomy, Department of Veterinary Medicine, FU Berlin; Scores were given on a 7-point Likert scale; s=standard deviation.

**Figure 3:**
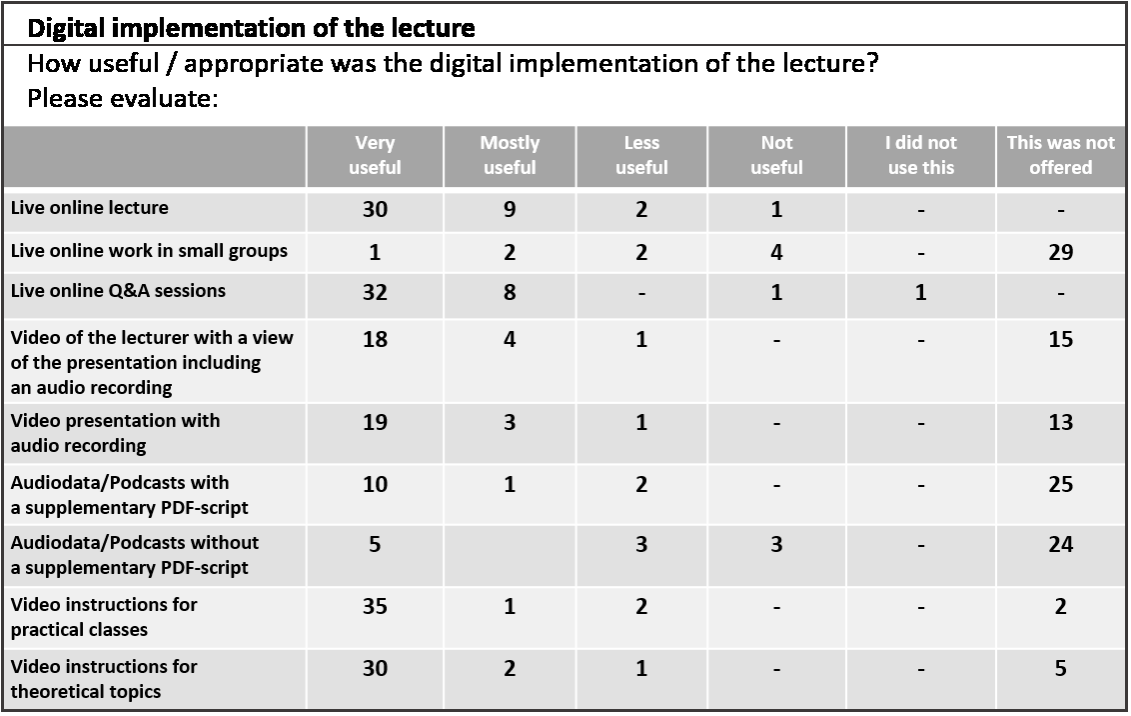
Partial results from student evaluations (N=51, 4^th^ semester) regarding digital teaching material in the summer term 2020, Institute of Veterinary Anatomy, University of Veterinary Medicine Hannover, Foundation.

All educational institutions encouraged students to evaluate their teaching efforts at the end of the semester. Results demonstrated that most students felt being supplied with a sufficient amount of learning material (see figure 2 (Fig. 2) and figure 3 (Fig. 3)). However, many students emphasized that practical classes were irreplaceable for understanding the complex anatomy and orientation of animal bodies in the evaluation’s suggestions box. Aside from the practical classes, students also missed the social interaction with their teachers. Subsequently, regular online office hours with lecturers were held to address this need, starting in the winter term. This semester was planned as a hybrid semester at all educational institutions, combining digital teaching (synchronous/asynchronous) and practical classes in small groups. This format came with a much higher personnel and material cost compared to “pre-COVID-19” teaching. 

The five educational institutions do not share the same anatomy curriculum, which means that the same content is taught at different time points throughout the preclinical years. This could explain why the institutions had not coordinated and shared their teaching efforts previously. Despite initial concerns, it was possible to share and use teaching material via the digital infrastructure of the creating institutions, which made teaching under these circumstances much easier. All digital materials that were created before or during the summer term will remain accessible to students as additional learning material after the pandemic and lay the foundation for a continuously growing joint resource pool. Nevertheless, lecturers are convinced that the practical classes in anatomy, histology and embryology, as specified by the degree regulations, are essential for students, as they provide tomorrow’s veterinarians with the practical knowledge and skills they need to successfully progress into the clinical semesters. 

## Competing interests

The authors declare that they have no competing interests.
